# Exploring long-term follow-up of effective implementation trials in schools: a secondary review

**DOI:** 10.3389/fpubh.2026.1725392

**Published:** 2026-02-02

**Authors:** Carly Gardner, Alix Hall, Cassandra Lane, Alison Zucca, Sam McCrabb, Edward Riley-Gibson, Xiao Tian Loh, Katherine Farragher, Rachel Sutherland, Nicole Nathan

**Affiliations:** 1 School of Medicine and Public Health, Faculty of Health and Medicine, The University of Newcastle, Newcastle, NSW, Australia; 2 The National Centre of Implementation Science (NCOIS), The University of Newcastle, Newcastle, NSW, Australia; 3 Hunter Medical Research Institute, New Lambton Heights, NSW, Australia; 4 Hunter New England Population Health, Hunter New England Local Health District, Wallsend, NSW, Australia; 5 Food and Nutrition Research Program, Hunter Medical Research Institute, New Lambton Heights, NSW, Australia

**Keywords:** chronic disease prevention, EBI sustainment, effective implementation interventions, health promotion, implementation science, schools

## Abstract

**Background:**

To reduce chronic diseases, evidence-based health promotion interventions (EBIs) must be effectively implemented and sustained in settings such as schools. This review assessed the extent to which EBIs sustained their effects following the completion of an effective implementation trial. It also explored the use of recommended sustainment practices in follow-up studies.

**Materials and methods:**

A Cochrane systematic review served as the basis for identifying school health promotion EBIs with demonstrated implementation effectiveness. Eligible studies were controlled trials in elementary or secondary schools that evaluated an implementation intervention and reported a statistically significant implementation effect. Forward citation searches were conducted across three electronic databases and two trial registration databases to identify relevant follow-up studies. To be included, follow-up studies needed to report comparable quantitative data (i.e., similar samples and outcome measures) to the original trial and to have been collected at least 6 months after the intervention ended. Two independent reviewers screened studies and extracted data using a predetermined template, with input from a third reviewer as needed. The included papers were assessed against sustainment-promoting practices, including whether they planned for, defined, or used a sustainment framework. The percentage of the original implementation effect sustained at follow-up was calculated.

**Results:**

Of the 23 EBIs with a significant implementation effect in the original review, 26% (*n* = 6) were found to have eligible follow-up studies. These targeted physical activity (*n* = 3), diet (*n* = 2), and tobacco prevention (*n* = 1). Four studies reported implementation outcomes, and two reassessed behavioral outcomes. The percentage of the implementation effect sustained ranged from 51 to 122%, with a median of 76% (IQ1 = 56%, IQ3 = 107%).

**Conclusion:**

To the best of our knowledge, this is the first review to quantify the sustainment of EBIs in schools following an effective implementation trial and to assess the extent to which best-practice sustainment principles were applied. Few studies described sustainment planning or used comparable follow-up measures. Improving long-term outcome measurement and integrating sustainment frameworks into planning and implementation could help sustain effective school health promotion EBIs.

## Introduction

Globally, chronic diseases account for approximately 75% of deaths each year (1). Many of the behavioral risk factors that contribute to these diseases, such as poor diet and physical inactivity, are established in childhood and track into adulthood (2). Schools are an important and convenient setting for promoting healthy behaviors in children because they offer consistent access to the majority of the population and reach children from diverse backgrounds who may face barriers to other health services. As such, schools are frequently tasked with delivering health promotion and evidence-based interventions (EBIs). Despite their potential, schools face several challenges when implementing health promotion EBIs, including competing curriculum demands, limited resources, and staff turnover (3, 4), which reduce the likelihood that programs will be implemented as intended, thereby undermining their effectiveness and long-term impact (5).

Over the past few decades, the field of implementation science has developed and tested strategies to address barriers that limit implementation (6, 7), including school-based health promotion EBIs (6–12). A 2022 Cochrane Systematic Review identified 38 studies evaluating strategies to improve the school-based implementation of EBIs targeting healthy eating, physical activity, weight gain, tobacco use, or alcohol use. Of these, 22 were randomized controlled trials (RCTs) that demonstrated significant positive effects on implementation outcomes [standardized mean difference (SMD): 1.04, 95% CI: 0.74–1.34; 1,917 participants]. While these findings highlight the potential for implementation support to improve adoption, their value depends on whether schools continue to deliver the EBI once active implementation support ends, a process often referred to as sustainment (13). Without sustained delivery, initial implementation gains are unlikely to translate into enduring public health outcomes.

To increase the likelihood that EBIs are sustained after implementation support ends, researchers have identified several practices to consider (14–18). These include (i) planning for sustainment during intervention and implementation design phases (14, 15, 17), (ii) explicitly defining the outcomes or domain(s) expected to continue, that is, delivery of the EBI, behavior change or use of implementation supports (14, 17, 18), (iii) applying sustainment specific-frameworks to guide both design and evaluation (13, 17), and (iv) reporting the type, duration, and intensity of any ongoing support provided after active implementation ends (19). Despite their potential to inform sustainability, these practices are rarely applied. A 2019 review of 26 community-based public health EBIs reporting sustainment outcomes found only 19% used a sustainment framework, and 27% provided an explicit definition of sustainment (18). Without consistent application of these practices, it is difficult to identify what supports EBI sustainment or to enable comparison across settings.

Despite growing recognition of its importance, sustainment is rarely assessed in school-based implementation trials. This limits our understanding of the long-term delivery or ongoing health impact of effectively implemented EBIs in school settings. Sustainment research across public health programs indicates it is important to consider both program continuation (sustainment) and the maintenance of behavioral outcomes when exploring a program’s continued benefit (18).

Where follow-up in the school setting has occurred, findings suggest that both EBI delivery and behavioral outcomes often decline once implementation support ends. For example, a 2020 review by Herlitz et al. examined the extent to which school-based EBIs were sustained after initial delivery (20). The review found none of the 18 included EBIs sustained all intervention components; however, 17 EBIs continued at least one component in some schools. While valuable, this review included EBIs regardless of whether they were implemented effectively (demonstrated a positive effect on an implementation measure). Evaluating the sustainment of EBIs without first confirming implementation success risks drawing misleading conclusions about what supports long-term delivery, as sustainment cannot occur without initial implementation. To best inform future efforts, evidence on sustainment should focus on EBIs that have demonstrated successful implementation in real-world conditions (15).

To date, no review has explicitly examined whether EBIs that have been effectively implemented in school settings have also been assessed for sustainment or whether recognized sustainment practices were applied. The 2022 Cochrane review by Wolfenden et al. (21) provides an ideal sampling frame for such an investigation, offering a curated set of EBIs that have been shown to be successfully implemented following an effective implementation intervention. This secondary review builds on that work to:

1. Determine the proportion of school-based health promotion EBIs that assess sustainment (either program sustainment or sustainability of behavioral outcomes) after the conclusion of an effective implementation trial.
2. Identify if recommended practices of sustainment are present, including sustainment planning, defining sustainment, use of sustainment frameworks, use of sustainment strategies and level of support, and a threshold for program sustainment (e.g., 80% of sites continued to implement the EBI).
3. Assess the extent to which implementation effects are sustained following the end of an effective implementation trial.

## Materials and methods

This systematic review is reported in accordance with the Preferred Reporting Items for Systematic Review and Meta-Analysis Checklist (PRISMA) (see Supplementary file 1 for the completed checklist). Methods for conducting this review follow Cochrane best-practice guidance on systematic reviews (22).

### Eligibility criteria

All included studies from the Wolfenden review (21) were used as a sampling base. We acknowledge the sample review was updated in parallel to undertaking this review (23); however, the previous 2022 version was maintained as the sampling frame for the following reasons: (1) The newer edition restricts study eligibility to RCTs; however, RCT designs are uncommonly used for sustainment research, and thus we deemed the breadth in design eligibility in the 2022 version more appropriate for assessing sustainment; and (2) that additional EBIs identified (in the 2024 version) would unlikely have had time to publish subsequent follow-up studies aligned to our definition of sustainment (i.e., >6 months post implementation end). Studies were conducted in the school setting and targeted health promotion areas, including physical activity, obesity prevention, healthy eating, smoking or vaping cessation, and harmful alcohol use. Participant populations included staff, students, and parents involved in the schools in which studies were conducted.

To be eligible for inclusion, studies had to employ a parallel control group design that included RCTs or cluster RCTs (cRCTs); non-RCTs or cluster non-RCTs with a parallel control group; and controlled before-and-after studies (CBAs) or cluster CBAs.

Eligible trials were checked for a statistically significant implementation effect at the time point immediately following the implementation phase. When the original review reported outcomes at a different time point, the SMD was recalculated using data from the end-of-implementation time point. Statistical significance was taken from the paper (i.e., extracted from the paper) or calculated by the research team for the post-implementation effect (21). In cases where this could not be calculated (e.g., results not reported by the control and intervention groups), an EBI was included if it self-reported a statistically significant effect on the primary implementation measure at the end of implementation.

Those studies with a significant implementation effect were searched and screened for relevant long-term follow-up studies (defined as data accessible to us at a timepoint meeting the eligibility criteria described below, which could include the original trial paper, an additional publication, or data provided upon request). A follow-up study was included if it met the following criteria:

- Timepoint: Follow-up occurred ≥ 6 months after the end of implementation.
- Methods and study design: All quantitative study designs were considered, as previous research indicated that follow-up time points are infrequently conducted using controlled designs (24).
- Measure: Sustainment measured using a comparable implementation or behavioral outcome as reported at the end of implementation in the original study.
- Sample: Conducted with a comparable sample. This could be the same sample from the original implementation evaluation study or a new group that had received the effective implementation intervention using the same implementation strategies.

A follow-up study was excluded if:

- Timepoint: Follow-up occurred <6 months after the end of implementation
- Methods: Qualitative methods only
- Measure: Not comparable to the implementation effect study.
- Sample: Conducted with a new sample (e.g., at scale) but used different implementation strategies, not comparable to the implementation effect study, and thus the implementation effect is unknown.

Sustainment was reported based on the longest included time point post-implementation. It was classified using recommended categories from the literature (14, 17, 25, 26), that is, maintenance (6–12 months post-implementation), early sustainment (12–24 months post-implementation), and sustainment (>24 months post-implementation). ‘Post implementation’ was classified in the following ways (19).

- No ongoing support: The study outlined a timeline with a clear end date and described final data collection or implementation support strategies as ending.
- Lower than implementation support: The study described an intensive implementation support period as ending but outlined low-intensity support, such as access to a support officer (fewer counts than implementation), return to usual practice, or a periodic newsletter.

**Figure 1:**
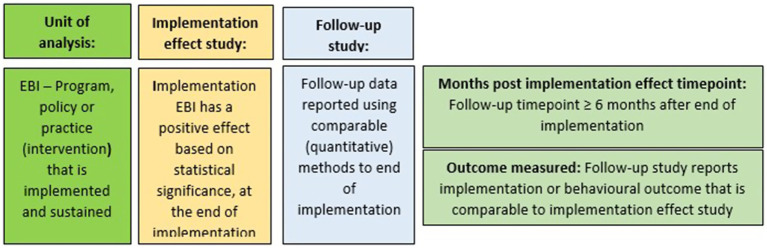
Explanation of unit of analysis and the inclusion summary.

There were no restrictions on country of origin, language, or publication date. To assess sustained implementation, the unit of analysis was the EBI. This approach allowed us to (a) incorporate previously reported trial information (e.g., pilot studies) for the use of sustainment frameworks, and so on and (b) allow for substitution: replacing the implementation effect study with a subsequent (eligible) trial that included a follow-up timepoint. As a result, multiple individual studies could be included for a single EBI. Figure 1 displays the unit of analysis and the corresponding studies (implementation effect/follow-up) that meet the inclusion criteria.

### Search strategy

#### Information sources

All citations relating to effective implementation trials (those with a statistically significant effect measured directly at the end of implementation) included in Wolfenden (2022) were used in the forward citation search.

#### Search process

We developed the following search strategy in consultation with an information specialist. Follow-up studies were sourced by the following:

1. Conducting a forward citation search in PubMed, Scopus, and Google Scholar for each effective EBI. Each citation associated with the EBI listed in the original review was searched (e.g., effect paper, pilot paper, process evaluation, and so on). Individual citations were searched in each search engine, and following ‘cited by’ or forward citation links, all listed studies were retrieved.
2. Searching the EBI registration listed on either the International Clinical Trials Registry Platform (ICTRP) or the relevant platform in the EBI country (e.g., Australian EBIs in the Australian New Zealand Clinical Trials Registry) sites
3. Searching the EBI name in Google for associated EBI websites, if available
4. Contacting the corresponding author of the original implementation at EBI via email if additional data is required

**Figure 2:**
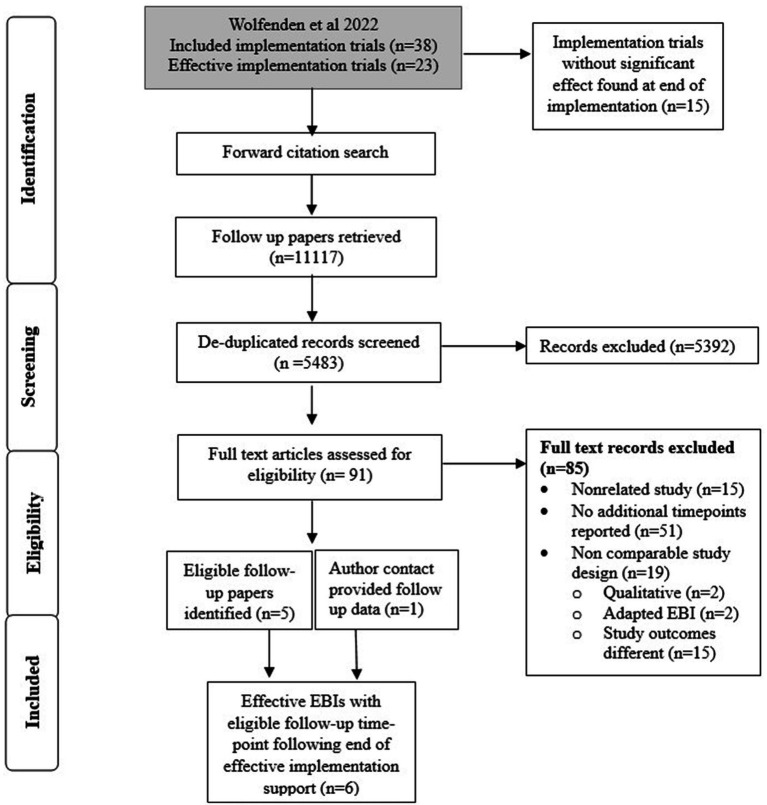
PRISMA flow diagram for included studies.

Screening began manually in EndNote, where each EBI was assigned to a group, and all citations identified during the search were collated and deduplicated. Citations were uploaded to Covidence, and title and abstract screening was conducted independently by two researchers (XL, CG). Two reviewers independently conducted full-text screening and recorded inclusion or exclusion in the online data capture tool REDCap (27) (CG, KF). Findings were cross-checked, and disagreements were resolved by consensus or by assessment by a third member of the research team (AH, AZ, NN). Search results are reported in the PRISMA flow chart (Figure 2).

#### Data collection process

Data extraction was conducted independently by two researchers using a piloted extraction form in REDCap (CG, KF). Any conflicting data was resolved by consensus or by a third member of the research team (AH).

### Data extraction

Data on EBI characteristics, implementation strategies, implementation behavioral outcomes, and sustainment characteristics were extracted at the EBI unit of analysis, using data from the Wolfenden review (21) and all eligible papers. The sustainment items selected for extraction are based on those identified by existing reviews and frameworks as pertinent to sustained EBI delivery (14, 15, 17, 28). A full-text version of the extraction template is available in Supplementary file 2.

- EBI characteristics: Author, year, EBI name (if one), country, setting, health behavior(s), trial design.
- Implementation strategies: extracted from Wolfenden (using EPOC framework) (29)
- End of implementation outcomes extracted from the Wolfenden, selected primary outcome reported in the review (21)
- Sustainment characteristics: Extracted items relating to sustainment were classified ‘yes/no’ and ‘occasionally’ with a section to describe sustainment planned for in design, definition of sustainment used, use of ‘sustainment’ or related term, use of sustainment-specific framework (author, date of framework), sustainment support (level of support and description), and threshold considered sustained (description).

### Effect measures

Where possible, the follow-up effect on implementation and behavioral outcomes was calculated using the same method as in the implementation effect studies (21). Specifically, for studies reporting a difference in outcomes between the control and intervention groups at long-term follow-up, we calculated SMDs and 95% confidence intervals to enable comparison across studies to assess the magnitude of the implementation intervention effect. This applied to papers reporting both continuous and dichotomous outcomes, using a standardized formula to convert to an SMD. For follow-up studies that reported implementation or behavioral outcomes for the intervention group only (i.e., no intervention vs. control comparison), we calculated the percentage of the outcome sustained at long-term follow-up. This indicated the percentage of the implementation effect that was sustained after an effective implementation trial ended. This follows methods used in other sustainability reviews that report sustainability as a percentage (14, 30) and in reviews assessing the effect of implementation at scale-up (31).

This was calculated as follows:


$$\frac{\text{Level ofoutcome}\;\mathrm{at}\;\text{long}-\text{term follow}-\mathrm{up}}{\text{Level ofoutcome after initial implementation}}\times 100$$

### Synthesis methods

For Aim 1, the percentage of effective implementation trials that assessed long-term sustainment was calculated. For Aim 2, sustainment characteristics were reported descriptively using a combination of yes/no indicators and text responses.

For Aim 3, a random-effects meta-analysis was initially planned to assess sustained implementation effects and sustained behavioral outcomes. However, this was not feasible due to the small number of studies reporting a difference in sustained implementation and/or effect on behavioral outcomes between the intervention and control groups at follow-up. Therefore, the median and interquartile range of the percentage of sustained effect across all EBIs were reported separately for implementation and behavioral outcomes to provide a broad indication of the extent to which school-based EBIs are being sustained following a successful implementation trial.

### Reporting bias assessment and critical appraisal

Quality assessments were conducted independently by two review authors (CG, ER-G) using Cochrane’s Risk of Bias tool version 1 (consistent with the Wolfenden review), and a third review author (AH) was consulted to resolve discrepancies. Risk of bias was assessed for all follow-up studies. The risk of bias for implementation effect studies was previously assessed in the original review (21). Judgments made in the original review were extracted and included in this review, where follow-up studies used the same design and sample as the implementation trial (domains include selection bias, performance bias, detection bias, recruitment to cluster, and baseline imbalance). Domains relevant to the follow-up timepoint were reassessed (attrition bias, reporting bias, loss of clusters, incorrect analysis, and potential confounding). Where follow-up studies used a different sample than the one used during EBI implementation, all domains were reassessed. “Overall assessment of bias” was reassessed for all EBIs. Risk of bias was reported as “high,” “low,” or “unclear” as per the Cochrane Handbook of Systematic Review of Interventions (32). Where follow-up studies used non-controlled designs, the Newcastle-Ottawa Quality Assessment Scale (NOS) was used, specifically the adapted version for cross-sectional studies, which has been applied in other reviews (33, 34). The NOS rates the quality of studies with a star system, assigning a maximum of 10 stars for the cross-sectional study tool. Three domains were assessed: selection of study groups, comparability of groups, and assessment of outcomes.

## Results

### Search

Of the 38 included implementation trials in the Wolfenden (2022) review, 23 reported a significant implementation effect and were eligible for this forward citation search (21). After deduplication, a total of 5,483 individual studies were generated through the forward citation search (Figure 2). A total of 91 studies were screened for full text, leading to the identification of six EBIs with associated follow-up papers for inclusion. In most cases, excluded papers (*n* = 85) did not report data from a follow-up time point (*n* = 51); instead, they typically reported data at implementation time points (e.g., process evaluations or subgroup analyses). The search identified a follow-up time point for one of the eligible studies, collected during a later cRCT implementation. This updated data replaced the implementation effect trial included in the Wolfenden (2022) review, as the newer trial used comparable implementation strategies and outcomes (8, 35). The included follow-up papers were grouped with their corresponding implementation-effect studies and reported in Table 1.

**Table 1:** Characteristics table of included implementation-effect studies and corresponding follow-up articles.

Primary author, year, *EBI name* (if one), country	Setting and targeted health behavior(s)	Implementation trial design (sample size)	Implementation outcome	Implementation strategies (EPOC) (29)	Implementation effect SMD [CI]	Follow-up paper, author (year)	Follow-up study design (sample size)	Time since implementation ended (months)
EBIs with eligible follow-up timepoint *n* = 6								
Implementation outcomes reported at follow-up								
Nathan et al., 2022, *Physically Active Children in Education (PACE)*, Australia (8, 35)	Elementary schools (kindergarten – 6th grade), Physical Activity	cRCT (61 schools)	Teacher implementation of physical activity policy across a school week (minutes)	Education materials Educational outreach visits or academic detailing Local opinion leaders Tailored interventions Other: mandate change	0.65 [95% CI 0.21 to 1.10]	Nathan et al. (2022) (8)	cRCT (61 schools)	6 months
Wolfenden et al., 2017, *Canteens*: (*no formal name*), Australia (53)	Elementary schools, nutrition	cRCT (70 schools)	Healthy items represented >50% of the canteen menu (more is better)	Audit and feedback Continuous quality improvement External funding Educational materials Educational meeting Educational outreach visits Local consensus process Local opinion leader Tailored intervention Other: newsletter, acknowledgment of compliant schools, kitchen equipment supplied	1.37 [95% CI 0.68 to 2.07]	Wolfenden et al. (2019) (37)	cRCT (56 schools)	12 months
Sallis et al. 1997, *Sports, Play, and Active Recreation for Kids (SPARK),* United States (42)	Elementary schools (grades 4–5), PA	Non RCT (seven schools) – two intervention arms: teacher trained; two PE specialist	Amount of physical education per week (minutes) (more is better)	All schools (including control): Other: equipment supplied All intervention schools: Educational materials Length of consultation Teacher trained arm only: Educational meetings Educational outreach visits	1.10 [95% CI 0.55 to 1.64]	McKenzie (1997) (39)	Experimental, (*n* = 14 teachers)	18 months
Nathan et al. (2012). *Good for Kids. Good for Life*, Australia (40)	Elementary schools (kindergarten – 6th grade), nutrition	Non-RCT (828 schools)	Prevalence of vegetable and fruit breaks	Local consensus process Local opinion leaders Educational meetings Educational materials Tailored intervention Monitoring the performance of the delivery of health care Other: Incentives	0.59 [95% CI 0.32 to 0.86]	The author supplied data	Cross-sectional survey (15 schools)	120 months
Behavioral outcomes at follow-up								
Mathur et al. (2016), *Bihar School Teachers Study*, India (41)	Government high schools (grades 8–10), Tobacco cessation and prevention	cRCT (72 schools)	Cessation of tobacco products among teachers and implementation of school tobacco policies.	Local opinion leaders Continuous quality improvement Educational meetings Educational materials Local consensus process	1.11 [95% CI 0.88 to 1.34]	Sorensen (2013) (36)	cRCT, follow-up paper (72 schools, 677 employees)	9 months
Farmer et al., 2017, *PLAY*, New Zealand (38)	Government elementary schools, Physical Activity	cRCT (16 schools)	Play space evaluation score	Audit and feedback Local consensus process Tailored interventions Other: external funding	1.2 [95% CI 0.13 to 2.26]	Farmer et al. (2017) (38)	cRCT (16 schools, 669 students)	12 months

EBI, evidence-based intervention; cRCT, cluster randomised controlled trial; EPOC, Cochrane Effective Practice and Organisation of Care (EPOC) taxonomy; SMD, standardized mean difference.

### Characteristics of included studies

**Figure 3:**
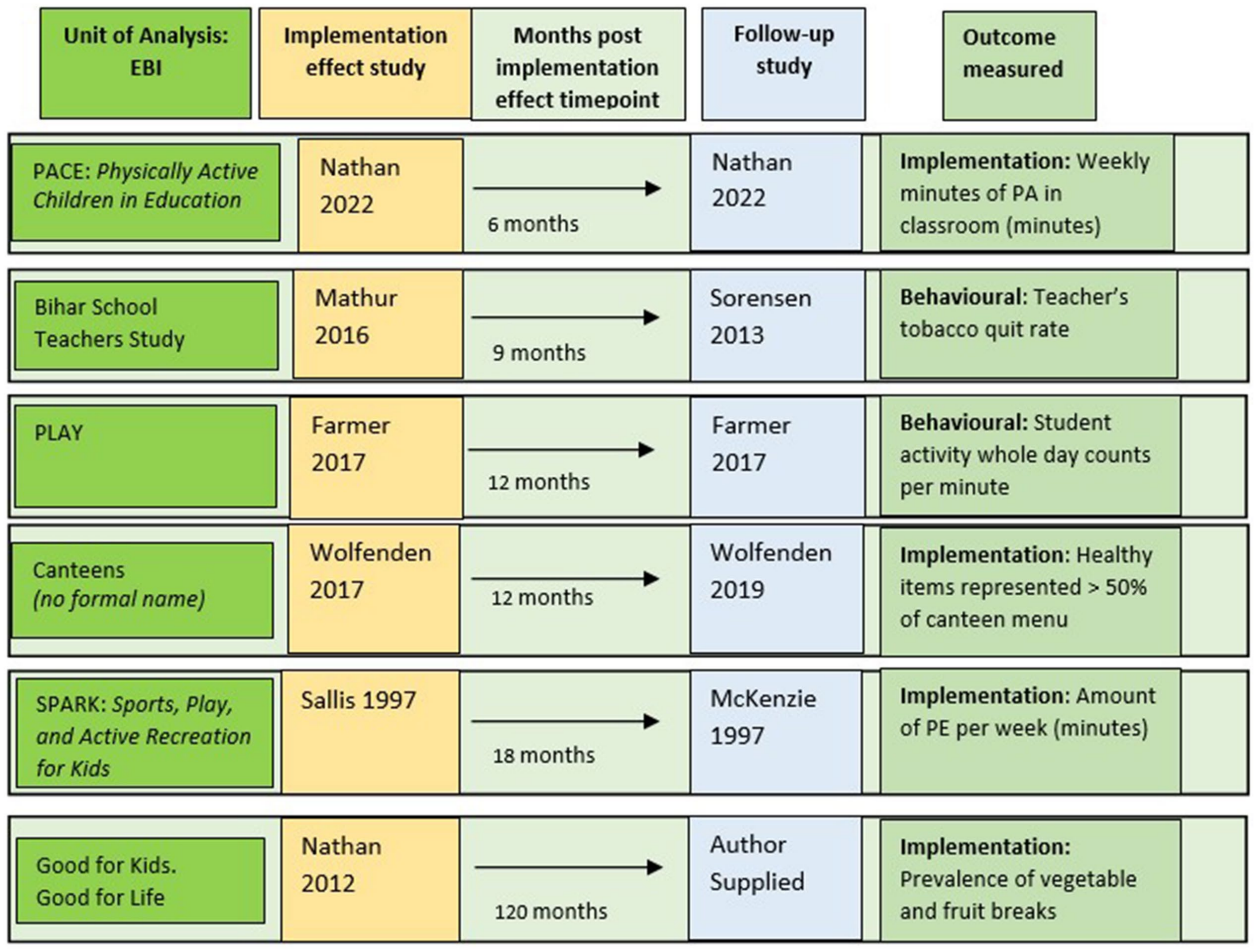
Summary of included EBIs, including papers used, timepoints, and outcomes measured at follow-up.

As shown in Table 1, two follow-up studies collected data 6–12 months after implementation ended (8, 36) (classified as maintenance), and three between 12 and 24 months (37–39) (classified as early sustainment). One was conducted longer than 24 months (personal communication, September 9, 2024) (classified as sustainment). Most included EBIs had been delivered to students and staff in elementary schools (8, 37–40), with one in the high school setting (36). Three interventions aimed to increase student physical activity (8, 38, 39), two targeted dietary behaviors (37, 40), and one aimed to prevent and cease tobacco use (36). While all implementation effect studies included a control comparison, only two follow-up studies reported implementation outcomes with a comparison group (8, 37), and a further two reported behavioral outcomes by comparison group (36, 38), but not implementation outcomes. Two reported follow-up data for intervention groups only (39, 40). The EBIs, including studies and data collection time points, are summarized in Figure 3.

### Quality assessment

**Figure 4:**
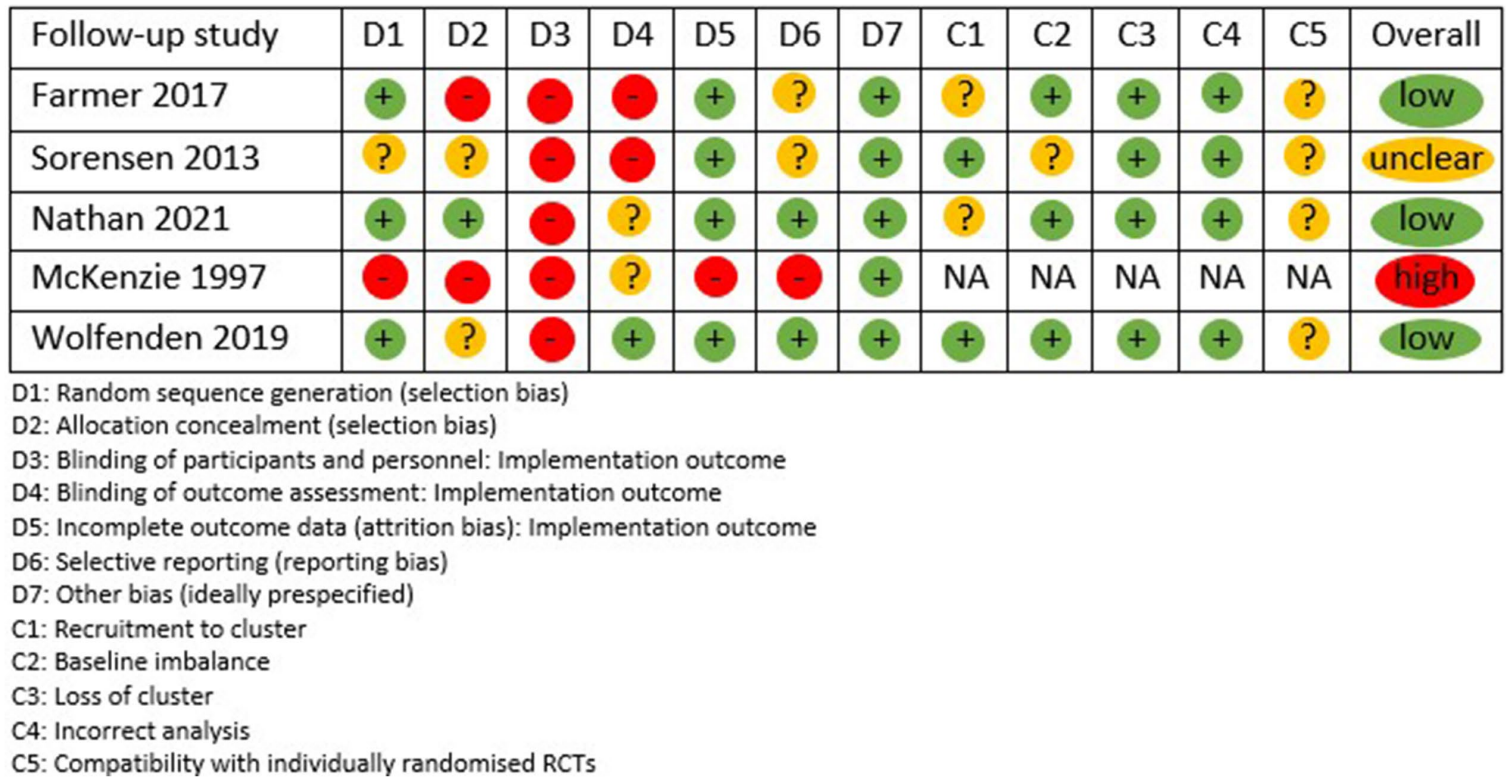
Risk of bias.

**Figure 5:**
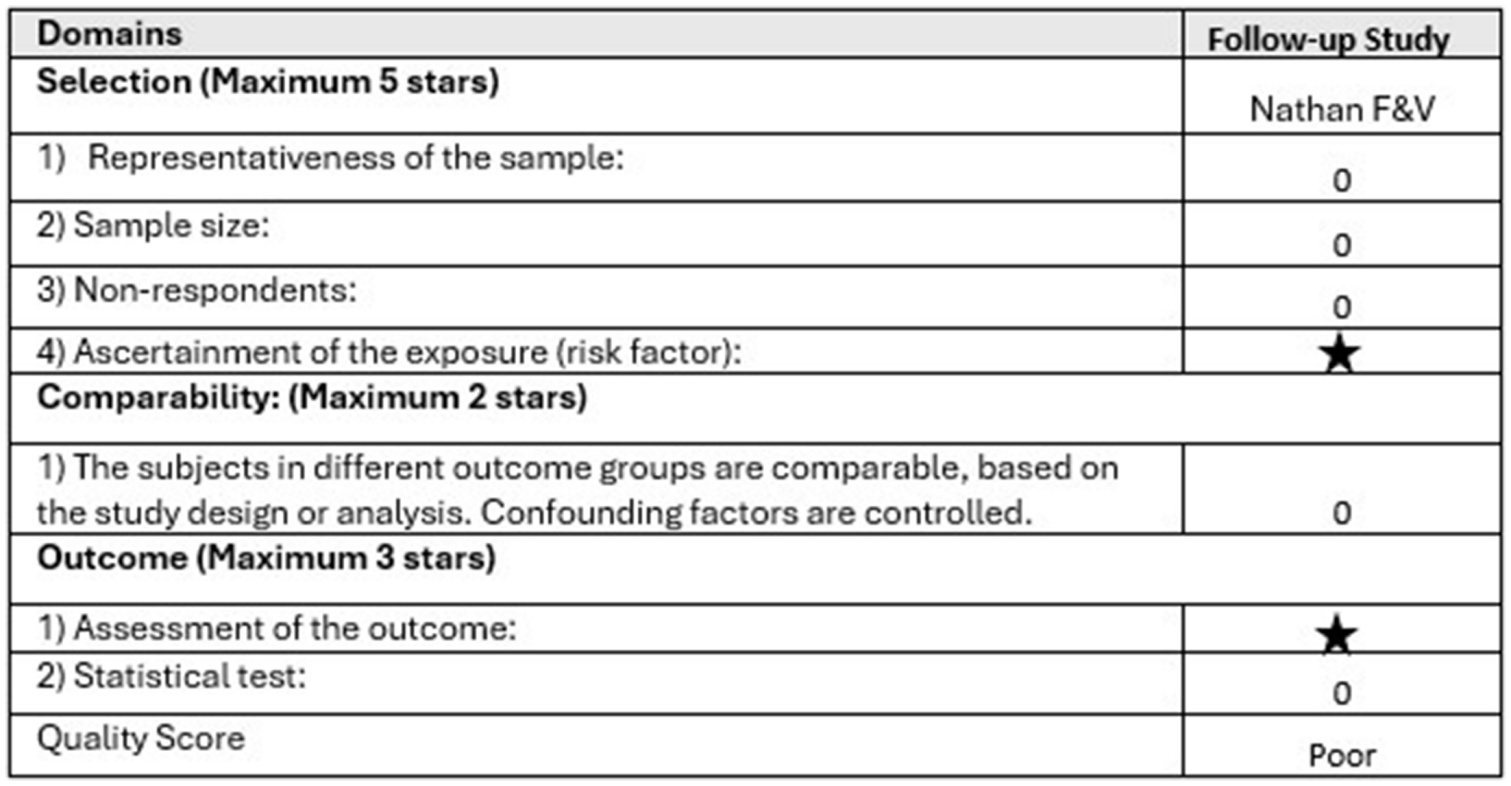
Newcastle Ottawa Scale quality assessment.

Risk of bias was used to assess the quality of five included follow-up studies shown in Figure 4, and one study was assessed using the NOS shown in Figure 5. Three studies were deemed low risk, two were deemed high risk or low quality, and one risk was unclear. The highest-risk-of-bias domains relate to blinding of participants and personnel during outcome implementation and to blinding of outcome assessment, reflecting the nature of the trials, where blinding is difficult to achieve.

### Aim 1: percentage of EBIs that assess sustainment after the conclusion of an effective implementation trial

Six of the 23 (26%) implementation effect studies collected comparable follow-up data. Sustained implementation was assessed by four (17%), and maintenance of the EBI’s behavioral outcomes was assessed by two (9%) at a long-term follow-up time point.

### Aim 2: presence of recommended sustainment practices reported by EBIs

As shown in Table 2, four of the six included EBIs were planned for long-term follow-up, as reported in the implementation effect study (8, 38, 41), or through program monitoring with repeated cross-sectional surveys (40). Two used “sustainment” or related terms in the original EBI papers (8, 41), and one of these defined sustainability as “*the extent to which a newly implemented treatment is maintained or institutionalized within a service setting’s ongoing, stable operations*” (8). No studies followed a sustainment-focused framework or considered a threshold for sustainment. While no studies reported providing ongoing implementation support, one EBI (8) stated that all participant schools were able to access resources and phone support with a health department officer during the maintenance period; hence, the classification as providing ongoing support at a level ‘lower than implementation support’.

**Table 2:** Presence of sustainment practices (definition and so on) in follow-up studies, reported by the sustainment period.

EBI (follow-up paper author, year)	Long-term follow-up planned Y/N (description)	Sustainment defined Y/N (reference)	Sustainment term used in original papers Y/N, term used (paper type, author, year)	Sustainment framework or theory used Y/N	Level of external support at the sustainment follow-up time point	Strategies reported at follow-up
Studies reporting at maintenance timepoint (6–12 months)						
PACE (Nathan, 2022) (8)	Y (planned 6-month follow-up in this paper)	Y (Proctor et al.) (16)	Y, sustainability (Protocol, Nathan, 2019)	N	Lower than implementation	Online resources, telephone support
Bihar School Teachers Study (Sorensen 2013) (36)	Y (planned 6-month follow-up in implementation effect study)	N	Y, maintenance, (outcomes, Mathur, 2016) (41)	N	No ongoing support	None reported
Studies reporting at early sustainment timepoint (12–24 months)						
PLAY (Farmer 2017) (38)	Y (planned 12-month follow-up in this paper)	N	N	N	No ongoing support	None reported
Wolfenden (Wolfenden 2019) (37)	N (long-term follow-up not mentioned in papers)	N	N	N	No ongoing support	None reported
SPARK (McKenzie 1997) (39)	N (long-term follow-up not mentioned in papers)	N	N	N	No ongoing support	None reported
Studies reporting at the sustainment timepoint (24 months or greater)						
Nathan V&F (author supplied data 2024)	Y, repeated cross-sectional surveys (correspondence)	N	N	N	No ongoing support	None reported

### Aim 3: level of effect sustained in relation to the implementation outcome/s following an effective implementation trial

Of the six studies that conducted a longer-term follow-up, four were RCTs (8, 37, 38, 41) and two were non-RCTs (42). Regarding the sustainability outcomes assessed, four assessed the long-term implementation effect (8, 37–39), and two assessed the long-term behavioral effect only (36, 38). Table 3 reports the sustained effect of the measured outcome.

**Table 3:** Percentage of outcome effect sustained at the follow-up time point following the end of an effective implementation intervention.

Timepoint	Both	End of implementation	Follow-up	Follow-up	End of implementation	Follow-up	Follow-up
EBI (follow-up author year) (RoB)	Outcome measure used	Outcome effect (M) SMD [CI]	Length of follow-up (months)	Follow-up outcome effect (M)	Outcome in the intervention group	Outcome in the intervention group	% outcome sustained
Implementation outcome reported							
PACE (Nathan, 2021) 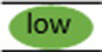	Weekly minutes of classroom physical activity	0.76 (0.56, 0.96)	6	0.46 (0.26, 0.66)	M = 148.5 (SD = 44.1)	M = 146.7 (SD = 40.3)	61%^b^
Wolfenden (37) 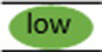	Healthy items represented >50% of the canteen menu (more is better)	1.38 [0.67, 2.09]	12	0.71 (−0.07, 1.49)	*N* = 22 (81.5%)	*N* = 24 (89%)	51%^b^
SPARK (McKenzie 1997) (39) 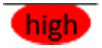	Amount of PE per week (minutes) (more is better)	1.10 [0.55, 1.64]	18	NR (no baseline data)^a^	M = 64.6 (CI 59, 70.2)	59.2 (SD = 27.5)	92%^a^
Nathan 2012 (40) (author supplied data) **/10	Prevalence of vegetable and fruit breaks at schools	0.59 [0.32, 0.86]	120	NR^a^	*N* = 318 (82%)	15 (100%)	122%^a^
Behavioral outcome reported							
Bihar Teacher Study (Sorensen 2013) (36) 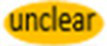	Tobacco quit rate	−0.25 [−47, −0.03]	9	−0.20 (−0.42, 0.0.02)	49.5% (with outcome)	17.4% (with outcome)	80%^b^
PLAY (Farmer, 2017) (38) 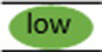	Student activity for the whole day counts per minute	−0.03 (−0.19, 0.13)	12	−0.12 (−0.28, 0.037)	M = 634 (SD = 164)	M = 566 (SD = 161)	−400%^b^

EBI, evidence-based intervention; RoB, overall risk of bias (reported in full detail in Figures 4, 5); PE, physical education; NR, not reported; M, mean; SMD, standard mean difference; CI, confidence interval.

a No reported difference between intervention and control at long-term follow-up; the effect at follow-up could not be calculated. Instead, the percentage sustained was calculated as the level of outcome achieved in the intervention group at long-term follow-up divided by the level of outcome achieved in the intervention group following initial implementation.

b The reported difference between intervention and control at both timepoints was calculated as the percentage of effect, defined as the SMD at long-term follow-up divided by the SMD following initial implementation.

#### Implementation outcome

Two of the follow-up studies reporting the long-term implementation effect employed an RCT design (8, 37) and compared the implementation effect between the control and intervention groups at follow-up. The SMDs for these two EBIs were calculated at the follow-up time point and are reported in Table 3. Both EBIs showed a reduction in implementation effect at long-term follow-up, as indicated by the between-group comparison. Across the four studies reporting sustained implementation effects, the percentage of the sustained effect ranged from 51 to 122%, with a median of 76% (IQ1 = 56, IQ3 = 107%).

#### Behavioral outcome

Two included EBIs reported behavioral outcomes at a follow-up time point after implementation ended, as shown in Table 3. The first (36) achieved 80% of the behavioral outcome effect 9 months after the implementation support ended. The other (38) reported a negative effect on the behavioral outcome at the end of implementation and found a further reduction in effect, four times the original effect, at the 12-month follow-up timepoint.

## Discussion

This review explored whether implementation trials that successfully improved the delivery of school-based health promotion EBIs also assessed longer-term sustainment and, if so, what level of sustainment was achieved. We leveraged existing data to explore the sustainment of school-based health promotion EBIs, an approach suggested by leaders in public health to maximize the value of prior investments and overcome challenges in sustainability research, such as resource constraints and the long timeframes typically required for follow-up studies (16). Of 23 effective implementation studies, we found only a small proportion (26%) conducted a comparable follow-up study, and just 4% assessed the delivery of the EBI beyond 24 months (16). Furthermore, data quality and comparability across follow-up studies were low, limiting the ability to draw firm conclusions on EBI sustainment. For example, several studies were excluded because they differed from the original implementation trial and the follow-up study, even though they appeared to assess sustainment (43, 44). Reasons for exclusion included different data types (e.g., quantitative vs. qualitative follow-up analyses) and the assessment of non-comparable outcomes or implementation strategies to those used in the original implementation trial. The lack of follow-up assessments, combined with limited data comparability, represents a missed opportunity to understand the full, long-term impact of implementation investments (17), an issue recognized in previous reviews on sustainment (24). To assess the impact of effective implementation on ongoing EBI delivery, the field needs to prioritize collecting and reporting comparable data (i.e., reassessing outcome measures for implementation and behavioral outcomes), ideally using controlled designs and at multiple time points, including at least 12 months post an effectively implemented EBI (16, 17, 24, 43–45).

We also examined whether the included EBIs applied practices that researchers have recommended for sustained EBI delivery. Consistent with previous reviews in public health settings, few studies included sustainment practices (14, 46) such as planning (*n* = 4), definitions (*n* = 1), or sustainment-specific frameworks (*n* = 0). This may be because sustaining EBI delivery was not the focus of these studies. These gaps may reflect when the studies were conducted, as the field has advanced in recent years. To improve future evidence, quality sustainment research should plan long-term assessments from the outset, draw on established sustainment definitions (18) and frameworks (13, 17), and use consistent outcome measurement to capture sustained implementation and behavioral outcomes (47). It is recommended that future research be undertaken to develop reporting guidelines for sustainment research that standardize the inclusion of these practices. This should help to improve the quality of future long-term follow-up studies and, in time, the ability to undertake rigorous evidence syntheses that improve our understanding of how to sustain effectively implemented programs (5, 17).

Positive health impact requires both sustained delivery of an EBI as well as maintenance of the positive impact the EBI has on behavioral outcomes (48). None of our included studies assessed both of these outcomes, with most (four out of six) focusing on implementation outcomes. Of the four that reported sustained implementation effects, two continued to show 90% or more implementation effects. In contrast, the other two EBIs reported results comparing intervention to control, with sustained effects of 51 and 61%. This could indicate program diffusion across the entire sample or other confounding factors, such as time or context. Across the four studies reporting implementation outcomes, 50% sustained effects at or above 80%, a rate more than twice that reported in previous public health sustainment reviews (14, 30), including in the school setting (20). Our observations are exploratory and limited by the small sample size and heterogeneity in outcomes; however, they suggest the potential for sustainment of effectively implemented EBIs (14, 20, 30). Theoretically, this makes sense, as EBI delivery cannot be sustained unless it is first successfully implemented, a hypothesis that aligns with implementation frameworks (48).

While continued delivery of EBIs is important for achieving population benefit, it is not sufficient on its own. The EBI itself must continue to exert a positive behavioral effect. In this review, we identified one study that found worsening behavioral outcomes at follow-up, with a follow-up effect four times lower than observed at the end of implementation, compared to control (38), despite a significant impact at the end of implementation. This emphasizes the importance of monitoring both continued program delivery and behavioral outcomes to assess ongoing impact at sustainment timepoints. This aligns with Moore et al.’s sustainability definition that includes constructs for continued program delivery, behavior change maintenance, and continued behavioral benefits (2017) (18). When program monitoring indicates that benefits are not continuing or have become harmful, guidance may be needed on how to improve the EBI or de-implement it (49). Importantly, to conduct reliable long-term EBI monitoring, we need increased research into developing and validating rigorous measurement tools that can address complexity and capture causal mechanisms in real-world program sustainment (47, 50).

### Strengths and limitations

We employed an efficient search strategy using a robust Cochrane systematic review as the sampling base. This approach leveraged EBIs already classified as implementation trials, enabling extensive, systematic searches of citations to identify related long-term follow-up papers. However, some eligible EBIs not included in the original review may have been overlooked. The majority of the included studies were from English-speaking, high-income countries and, as such, may have limited generalizability outside these contexts. The small sample is heterogeneous across target behaviors, design, outcome measures, and contexts, making interpretation challenging. However, it reflects the realities and challenges commonly faced in undertaking reviews in the area of implementation (14, 21). Furthermore, focusing on schools as the implementation setting provides consistency for this exploratory study, an approach used by other implementation-focused reviews (10, 21, 51).

Ideally, we would have calculated the sustained effect for all studies using data comparing control and intervention groups at long-term follow-up, but few reported adequate data. Instead, we relied on descriptive data from intervention groups in two studies and reported results for both behavioral and implementation outcomes. As a result, there is limited capacity to show whether between-group differences persist during the sustainment period. Despite this, we provide insights into sustained effects over time and highlight specific directions for improving reporting in the field.

An important strength of this study is its reporting of sustained EBIs following receipt of effective implementation interventions alone; however, this introduces potential selection bias, limiting the capacity to compare sustained effects between effective and ineffective trials. Similarly, publication bias may have affected follow-up data, with unfavorable results more likely to remain unpublished. To best understand sustained effects over time, it is important to publish long-term follow-up data regardless of outcomes.

## Conclusion

This review advances a priority in implementation science – understanding the long-term delivery of EBIs (52) – by examining whether effectively implemented health promotion EBIs in schools were sustained over time. Achieving EBI sustainment can yield sustained benefits for participants, enhance return on investment, and contribute to population-level health gains. However, if EBIs or their implementation are not effective, efforts to sustain them may be premature and potentially wasted. In such cases, resources should first focus on improving implementation fidelity and impact. Generating meaningful sustainment evidence requires long-term reporting of implementation outcomes from trials that demonstrate initial success. To achieve this, the field must prioritize appropriate resourcing and adequate timelines that support sustained follow-up (7, 16, 17). Failure to turn successful EBI implementation into sustained delivery represents a missed opportunity to yield maximum returns on implementation investments and will continue to fall short of population health impact.

## Data Availability

The data analyzed in this study are subject to the following licenses/restrictions: Data were extracted from published studies and summarized in this review; the extraction file is available from the authors upon reasonable request. Requests to access these datasets should be directed to carly.gardner@health.nsw.gov.au.
